# Sarco/Endoplasmic Reticulum Ca^2+^-ATPases (SERCA) Contribute to GPCR-Mediated Taste Perception

**DOI:** 10.1371/journal.pone.0023165

**Published:** 2011-08-02

**Authors:** Naoko Iguchi, Tadahiro Ohkuri, Jay P. Slack, Ping Zhong, Liquan Huang

**Affiliations:** 1 Monell Chemical Senses Center, Philadelphia, Pennsylvania, United States of America; 2 Givaudan Flavors Corporation, Cincinnati, Ohio, United States of America; Duke University, United States of America

## Abstract

The sense of taste is important for providing animals with valuable information about the qualities of food, such as nutritional or harmful nature. Mammals, including humans, can recognize at least five primary taste qualities: sweet, umami (savory), bitter, sour, and salty. Recent studies have identified molecules and mechanisms underlying the initial steps of tastant-triggered molecular events in taste bud cells, particularly the requirement of increased cytosolic free Ca^2+^ concentration ([Ca^2+^]_c_) for normal taste signal transduction and transmission. Little, however, is known about the mechanisms controlling the removal of elevated [Ca^2+^]_c_ from the cytosol of taste receptor cells (TRCs) and how the disruption of these mechanisms affects taste perception. To investigate the molecular mechanism of Ca^2+^ clearance in TRCs, we sought the molecules involved in [Ca^2+^]_c_ regulation using a single-taste-cell transcriptome approach. We found that Serca3, a member of the sarco/endoplasmic reticulum Ca^2+^-ATPase (SERCA) family that sequesters cytosolic Ca^2+^ into endoplasmic reticulum, is exclusively expressed in sweet/umami/bitter TRCs, which rely on intracellular Ca^2+^ release for signaling. Serca3-knockout (KO) mice displayed significantly increased aversive behavioral responses and greater gustatory nerve responses to bitter taste substances but not to sweet or umami taste substances. Further studies showed that Serca2 was mainly expressed in the T1R3-expressing sweet and umami TRCs, suggesting that the loss of function of Serca3 was possibly compensated by Serca2 in these TRCs in the mutant mice. Our data demonstrate that the SERCA family members play an important role in the Ca^2+^ clearance in TRCs and that mutation of these proteins may alter bitter and perhaps sweet and umami taste perception.

## Introduction

The sense of taste is important to animal survival and growth; it not only plays a critical role in determining what should be ingested but also generates hedonic valence that can significantly affect an animal's mood and mental activity [1]. Vertebrates, including humans, can recognize at least five primary taste qualities: sweet, umami, bitter, sour, and salty [2]. The interaction between the taste substances and receptors on the apical surface of taste bud cells initiates taste sensation, and the output signal from taste bud cells is relayed to the central nervous system via afferent gustatory nerves.

Each taste bud consists of a small number of basal cells, which are presumed to be progenitor cells, and a heterogeneous population of 50–100 elongated cells that are classified as type I, II, or III based on their morphological, biochemical, and physiological properties [3]. Type I cells are suggested to be salt-taste-sensing cells, in addition to the long-standing presumption of a supportive function in the taste bud similar to that of glial cells in the nervous system [4], [5], [6], [7].

Type II cells, also called taste “receptor” cells (TRCs), express molecular machinery for taste transduction for sweet, umami, and bitter, including G-protein-coupled taste receptors (GPCRs) and downstream effectors. Sweet and umami tastes are mediated by TRCs expressing GPCRs belonging to the T1R family: T1R2+T1R3 for sweet and T1R1+T1R3 for umami [8], [9], [10], [11], [12]. Bitter taste is mediated by another set of GPCRs, the T2R family [13], [14], [15], [16]. Activation of T1R or T2R receptors stimulates G-proteins, which in turn activate phospholipase C (PLC) β2, leading to the production of the second messengers inositol-1,4,5-triphosphate (IP_3_) and diacylglycerol [17], [18], [19]. IP_3_ binds to its receptor, IP_3_R3, allowing the endoplasmic reticulum (ER) to release Ca^2+^, which increases cytosolic free Ca^2+^ concentrations ([Ca^2+^]_c_), triggering the opening of Trpm5 (transient receptor potential cation channel, subfamily M, member 5) [17], [20], [21]. Na^+^ influx through Trpm5 depolarizes the membrane potential, which leads to release of ATP onto gustatory afferent nerve fibers via hemichannels [22], [23], [24].

Type III cells, also called “presynaptic” cells, form prominent synapses with afferent nerve fibers and exhibit depolarization-dependent secretion of serotonin and norepinephrine; these cells are also responsible for sour taste sensation [25], [26], [27], [28], [29], [30]. Although the signal transduction mechanisms for sour and salty taste qualities are not fully understood, studies have demonstrated that sour and salty taste stimuli also lead to increases in [Ca^2+^]_c_ in responding taste cells [5], [27], [31]. Therefore, regardless of the modality of a taste substance, the activation of different taste signal transduction pathways eventually converges at increases in [Ca^2+^]_c_, suggesting the importance of Ca^2+^ in taste signal transduction and transmission.

To utilize Ca^2+^ effectively as a signaling molecule, taste cells must tightly regulate [Ca^2+^]_c_ at both increasing and decreasing steps. Disruption of the exquisite [Ca^2+^]_c_ regulation in taste cells could cause nonspecific or inappropriate responses and, in severe cases, lead to cell death because of adverse effects of high [Ca^2+^]_c_ [32]. Thus, taste cells must have mechanisms to clear Ca^2+^ from cytosol to ensure the effectiveness of intracellular Ca^2+^ signaling mechanisms. To date, although many studies have focused on [Ca^2+^]_c_ elevation, Ca^2+^ clearance mechanisms in taste cells—how taste cells deplete Ca^2+^ from cytoplasm and restore [Ca^2+^]_c_ to prestimulation levels—are not well understood [17], [20], [33], [34], [35], [36]. Recent reports indicate that Na^+^/Ca^2+^ exchangers and mitochondrial Ca^2+^ transporters clear Ca^2+^ in presynaptic taste bud cells that express voltage-gated Ca^2+^ channels (VGCCs) [34], [35], [37], [38], [39], [40], [41]. The Ca^2+^ clearance mechanisms utilized by other types of taste cells, however, remain to be determined; moreover, how Ca^2+^ clearance mechanisms affect taste detection *in vivo* needs to be characterized.

In the present study, we hypothesized that different types of taste bud cells may employ different mechanisms in the restoration of [Ca^2+^]_c_ following responses to taste stimuli to reset the cells for the next taste stimulus. To test this hypothesis, we generated and analyzed rat subtracted single-TRC cDNA libraries, and identified one of the sarco/endoplasmic reticulum Ca^2+^-ATPase (SERCA) family genes, *Serca3* (*Atp2a3*) [42], that is expressed exclusively in sweet/umami/bitter TRCs. Serca3-knockout (KO) mice showed altered behavioral and gustatory nerve responses to bitter substances. In addition, we also found that another SERCA family member, Serca2, is largely expressed in T1R3-expressing taste bud cells and may compensate for the Serca3 loss of function in the mutant taste buds. Taken together, our results illustrate that unique sets of Ca^2+^ clearance proteins are utilized by different types of taste bud cells, and that malfunction of these proteins can alter taste perception.

## Results

### Mammalian taste papillae express Serca3 cDNAs

To identify unique molecules possibly involved in [Ca^2+^]_c_ homeostasis in TRCs, we isolated individual taste bud cells from rat circumvallate (CV) and foliate papillae and reverse transcribed and amplified all transcripts from these single cells. A portion of the amplified single-cell transcriptomes was used for *post hoc* gene expression profiling by quantitative real-time PCR (qPCR) with primers listed in Table S1 for the following cell-type marker genes: *Entpd2* for type I cells, *Trpm5* for type II TRCs, *Tas1r1* for umami TRCs, *Tas1r2* for sweet TRCs, and *Snap25* for type III/presynaptic cells. Another portion of the remaining amplified products for TRCs was subtracted with cDNA for type I cells to facilitate the isolation of genes specifically or more abundantly expressed in TRCs than in type I taste bud cells. The subtracted cDNAs were further amplified, subcloned into plasmid vectors, and sequenced. A BLAST search of the insert DNA sequences against the GenBank database indicated that one clone, A14, completely matched a 0.3-kb segment of the 3′ end of the rat *Serca3a* sequence, a splice variant of sarco/endoplasmic reticulum Ca^2+^-ATPase 3 (*Serca3*/*Atp2a3*, GenBank accession number NM_012914.1) [42].

**Figure 1:**
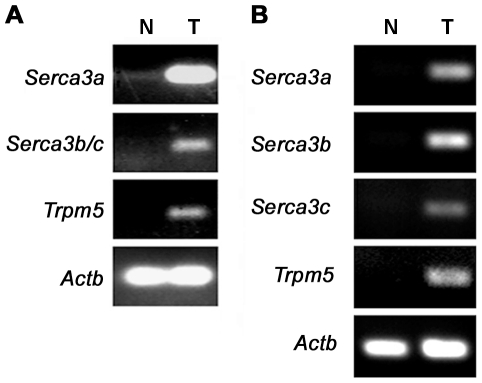
Selective expression of Serca3 splice variants in rat and mouse gustatory lingual epithelium. RT-PCR was performed with variant-specific primers, and RNA samples were prepared from rat (A) and mouse (B) taste-bud-containing papillae (T) or lingual epithelium devoid of taste buds (N). All known variants were detected. PCR reactions for Trpm5, a taste-specific marker, and β-actin (Actb), a general “housekeeping” gene, were included as controls.

Previous studies have shown that rodents and humans express splice variants that differ at the C-terminus: all variants except *Serca3a*, which is present in all human and rodent species, are species specific [43]. To investigate which isoform is expressed in rat and mouse taste tissue, we performed reverse-transcription PCR (RT-PCR) with variant-specific primer sets (Table S2). All known Serca3 isoforms—two in rats (*Serca3a* and *3b/c*) and three in mice (*Serca3a*, *3b*, and *3c*)—were detected in taste papillae (T), but not in lingual epithelium devoid of taste buds (N) (Fig. 1). To isolate the full-length coding cDNAs, sets of PCR primers were designed to cover the entire coding sequences (Table S2). PCR with the first-strand cDNAs from rat and mouse CV and foliate taste papillae, and subsequent insert DNA sequencing analysis, revealed no additional splice variants, and the full-length coding sequences for all mouse and rat isoforms except mouse Serca3c were detected.

### Sweet/umami/bitter TRCs express Serca3 proteins

**Figure 2:**
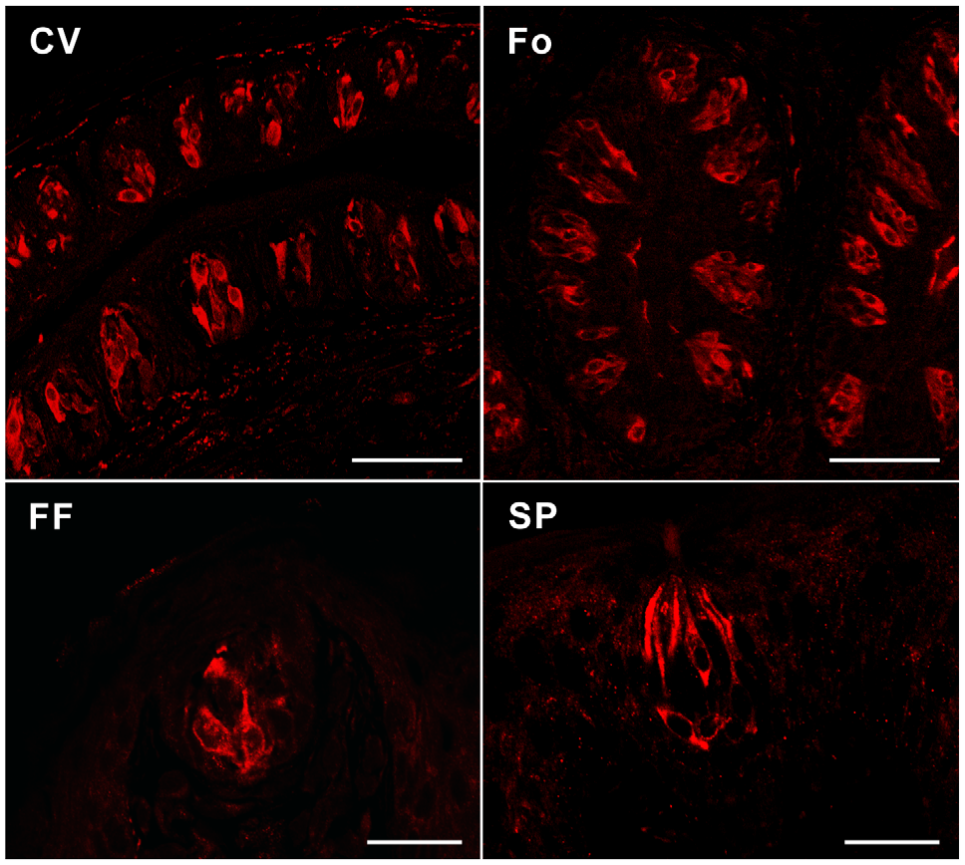
Expression of Serca3 protein in taste buds. Immunofluorescence images of mouse circumvallate (CV), foliate (Fo), fungiform (FF), and soft palate (SP) sections stained with the anti-Serca3 antibody show that immunoreactivity was detected in a subset of taste bud cells from all four major taste fields. Scale bars: 50 µm in CV and Fo, 25 µm in FF and SP.

To investigate Serca3 expression in taste papillae at the protein level, we performed immunohistochemistry against mouse lingual tissue sections with an antibody that recognizes all Serca3 isoforms. The robust signals were detected in a large subset of mouse taste bud cells obtained from four different oral taste fields (CV, foliate, and fungiform papillae and soft palate) (Fig. 2). Immunoreactivity extended throughout the cytoplasm of the labeled taste bud cells, which was not detected in taste tissues from Serca3 knockout (KO) mice, validating the specificity of the antibody as well as the absence of Serca3 proteins in the mutant mice (see below).

**Figure 3:**
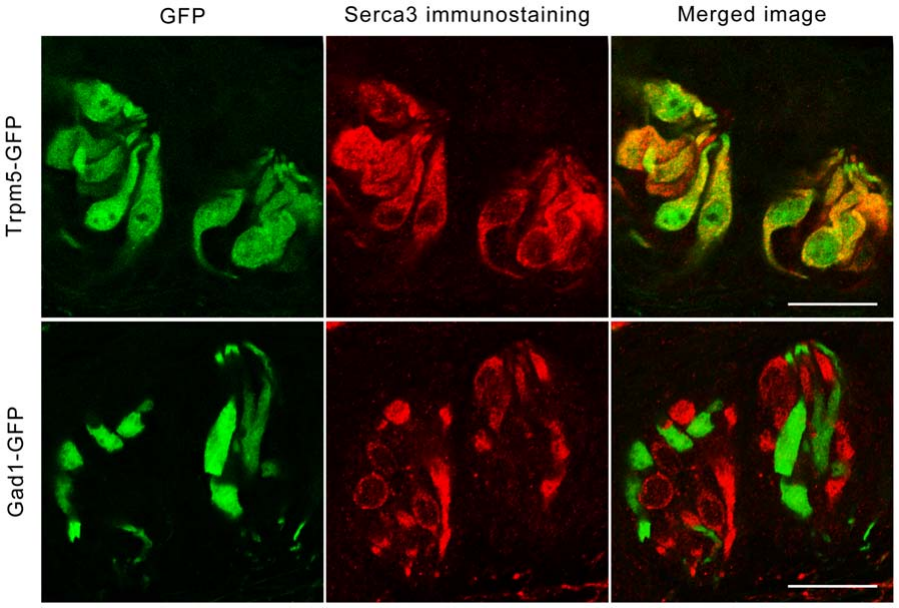
Serca3 protein is found in Trpm5- but not Gad1-expressing cells. Confocal images of CV taste papillae sections show the fluorescence of GFP(green, left) expressed under the control of Trpm5 (upper panels) or Gad1 (lower panels) promoters, labeling sweet/umami/bitter TRCs and most presynaptic cells, respectively, and the immunostaining with the anti-Serca3 antibody (red, center panels). The merged images show near-perfect overlap (yellow-orange) in Trpm5-GFP mice (upper right) but no overlap in Gad1-GFP mice (lower right). Scale bars, 25 µm.

Next, we explored which type of cells express Serca3 protein in taste buds by using anti-Serca3 antibody to immunostain taste papillae from two different transgenic mouse lines that expressed green fluorescent protein (GFP) under the promoter of either Trpm5 (Trpm5-GFP) or glutamic acid decarboxylase 1 (Gad1-GFP). The GFP expression of these two transgenic lines in sweet/umami/bitter TRCs and in the majority of presynaptic cells, respectively, was previously verified [31], [40], [44], and further confirmed by our immunocytochemical studies with antibodies against PLCβ2, Trpm5, and dopa decarboxylase (DDC), another marker for presynaptic cells [17], [45], [46] (data not shown). We found nearly perfect overlap of cell populations exhibiting GFP fluorescence and Serca3 immunoreactivity in the Trpm5-GFP mice, suggesting that Trpm5 and Serca3 are fully co-expressed in TRCs (Fig. 3, upper panels). In the contrast, Serca3 antibody staining on taste tissue sections of Gad1-GFP transgenic mice displayed mutually exclusive patterns (Fig. 3, lower panels), indicating that Serca3 is not expressed in the presynaptic cells. Taken together, these results show the specific expression of Serca3 in TRCs, suggesting a possible role for Serca3 in sweet/umami/bitter taste sensation.

### Normal morphology and expression of taste-related proteins in Serca3 KO taste tissues

**Figure 4:**
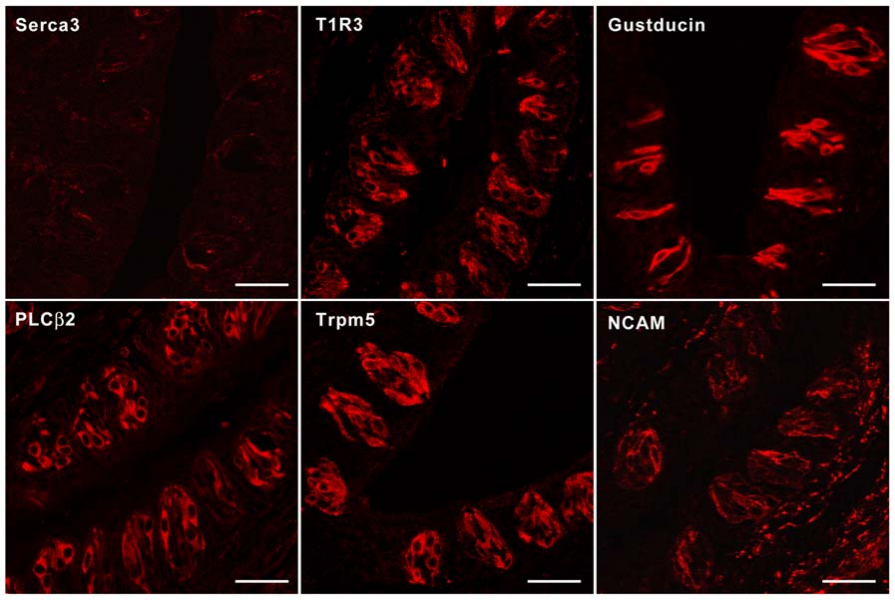
Normal taste bud morphology and expression of taste-signaling molecules in Serca3 KO mice. Immunofluorescence images of Serca3-KO mouse circumvallate taste tissue sections with antibodies against Serca3, T1R3, α-gustducin, PLCβ2, Trpm5, and neural cell adhesion molecule (NCAM) indicate that Serca3 protein was absent in taste buds, whereas the expression of other proteins appeared unaltered. Scale bars, 25 µm.

To characterize the possible function of Serca3 in taste bud structure and physiology, we first examined the expression of several taste-related proteins and taste bud morphology in Serca3 KO mice [47]. The lingual epithelium and taste buds of the mutant mice appeared grossly normal. Immunostaining of the mutant taste buds with anti-Serca3 antibody detected no specific signals, verifying the lack of Serca3 protein expression in the KO mice. Immunohistochemical studies showed no obvious abnormalities in expression of the taste-signaling components T1R3, α-gustducin, PLCβ2, Trpm5, and neural cell adhesion molecule (NCAM) in the mutant taste buds (Fig. 4).

### Increased behavioral responses to bitter taste in Serca3 KO mice

**Figure 5:**
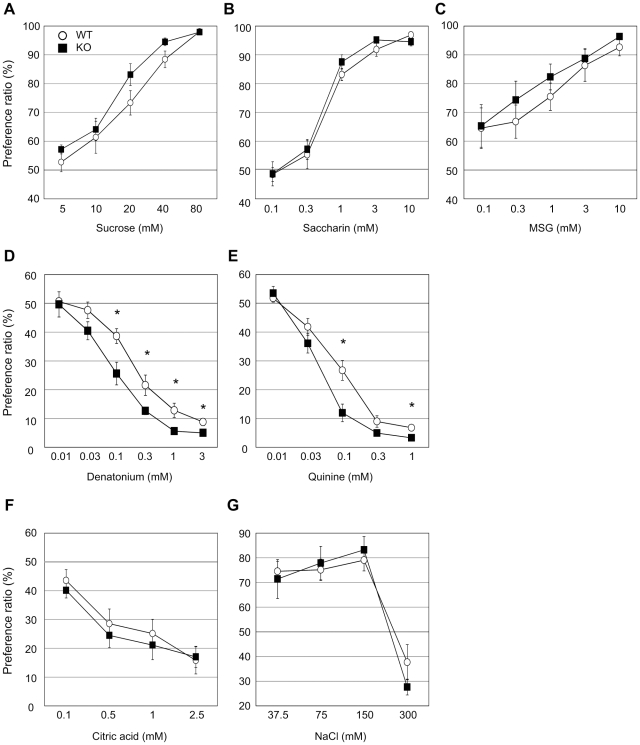
Mean taste preference ratios of WT and Serca3 KO mice using two-bottle preference tests. Taste responses are expressed as taste/water intake ratios of Serca3 KO (filled squares) and WT (open circles) to (A) sucrose, (B) saccharin, (C) monosodium glutamate (MSG), (D) denatonium, (E) quinine, (F) citric acid, and (G) NaCl. Data are means ± SEMs (n = 9–10, except n = 5 for KO mice for NaCl tests). Mixed-model ANOVA and *post hoc t*-tests were performed (*p<0.05).

To characterize behavioral responses of the Serca3 KO mice to tastants, we performed 48-h two-bottle preference tests with compounds representative of five primary taste qualities: sweet (caloric sucrose and the noncaloric artificial sweetener sodium saccharin), umami (monosodium L-glutamate or MSG), bitter (denatonium and quinine hydrochloride), sour (citric acid), and salty (NaCl). Data analysis indicated that there were no statistically significant differences between the wild-type (WT) and KO mice in the preference scores to sweet and umami taste stimuli, although the KO mice tended to display higher preference scores for the compounds at lower concentrations than did WT mice (Fig. 5A–C, Table S4). Interestingly, our data showed that mice deficient for Serca3 displayed significantly increased aversive responses to the bitter substances compared with WT mice (Fig. 5D and E, Table S4). We found no significant difference in the preference to ionic taste stimuli (sour and salty tastes) between the WT and KO mice (Fig. 5F and G, Table S4).

**Figure 6 pone-0023165-g006:**
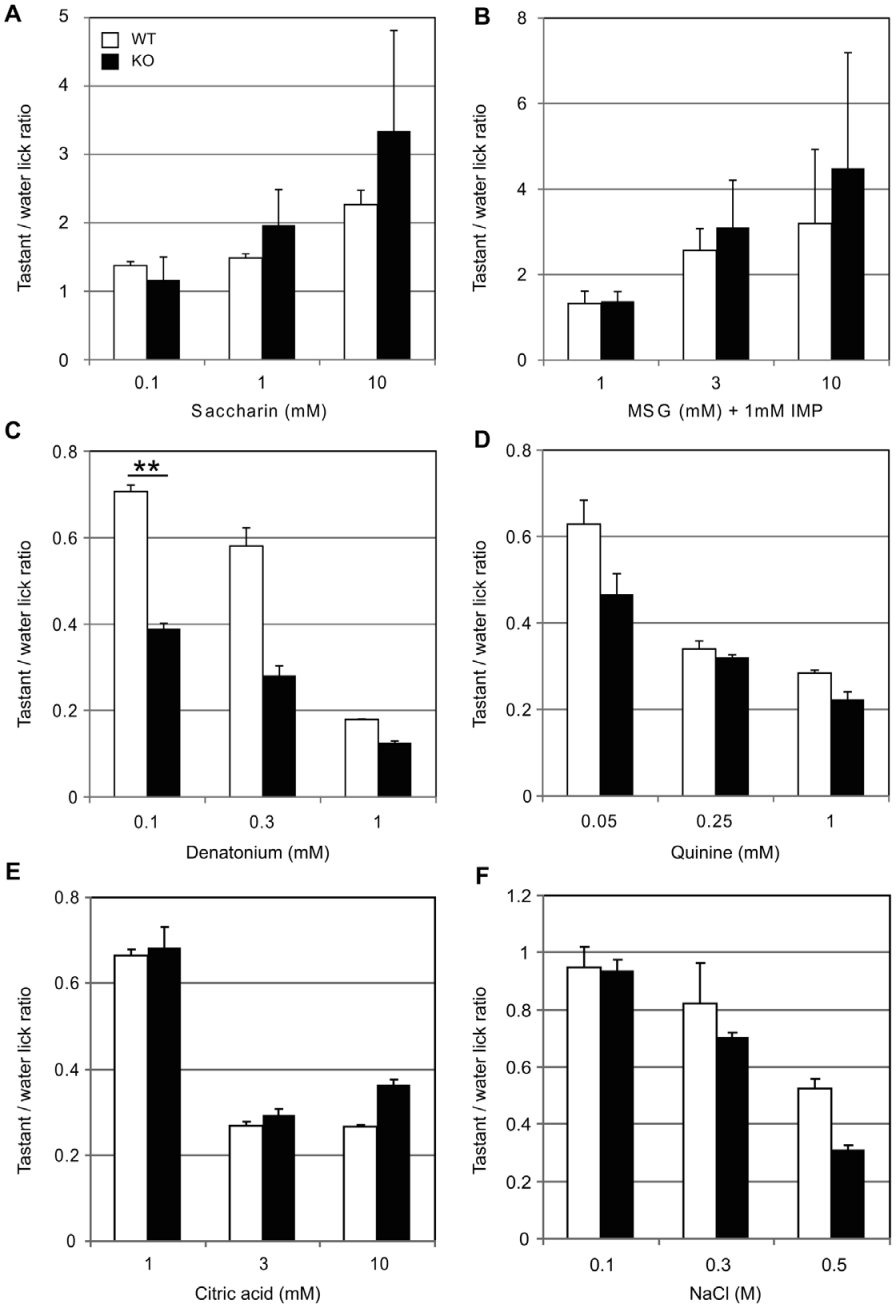
Altered taste responses of Serca3 KO mice in brief-access taste tests. Taste preferences of WT and Serca3 KO animals were measured relative to water using a brief-access taste test. Taste responses are expressed as taste/water lick ratios of Serca3 KO (filled bars) and WT (open bars) mice to (A) saccharin, (B) MSG in the presence of 100 µM amiloride and 1 mM IMP, (C) denatonium, (D) quinine, (E) citric acid, and (F) NaCl. Data are means ± SEMs (n = 7). Mixed-model ANOVA and *post hoc t*-tests were performed (**p<0.01).

We also carried out a brief-access procedure, which was designed to minimize possible postingestive effects. The results also showed that there were no significant differences between the responses of Serca3 KO and WT controls (n = 7 for each genotype) for the sweet and umami tastants (Fig. 6, Table S4), but there was a trend towards higher preference in KO mice for these palatable stimuli (Fig. 6A and B), as we observed in two-bottle preference tests (Fig. 5A–C). The behavioral responses of Serca3 KO mice to sour (citric acid) and salty (NaCl) taste substances were indistinguishable from those of the control WT mice (Fig. 6E and F). In contrast, KO mice displayed significantly increased avoidance of the bitter-tasting compound, denatonium at 0.1 mM, the lowest concentration we tested (Fig. 6C). For higher concentrations of denatonium (0.3 and 1 mM) and for another bitter-tasting substance, quinine (0.05, 0.25, and 1 mM), KO mice also seemed to exhibit increased avoidance responses, although the increases were statistically insignificant (Fig. 6C and D, Table S4).

### Increased gustatory nerve responses to bitter tastants in Serca3 KO mice

**Figure 7:**
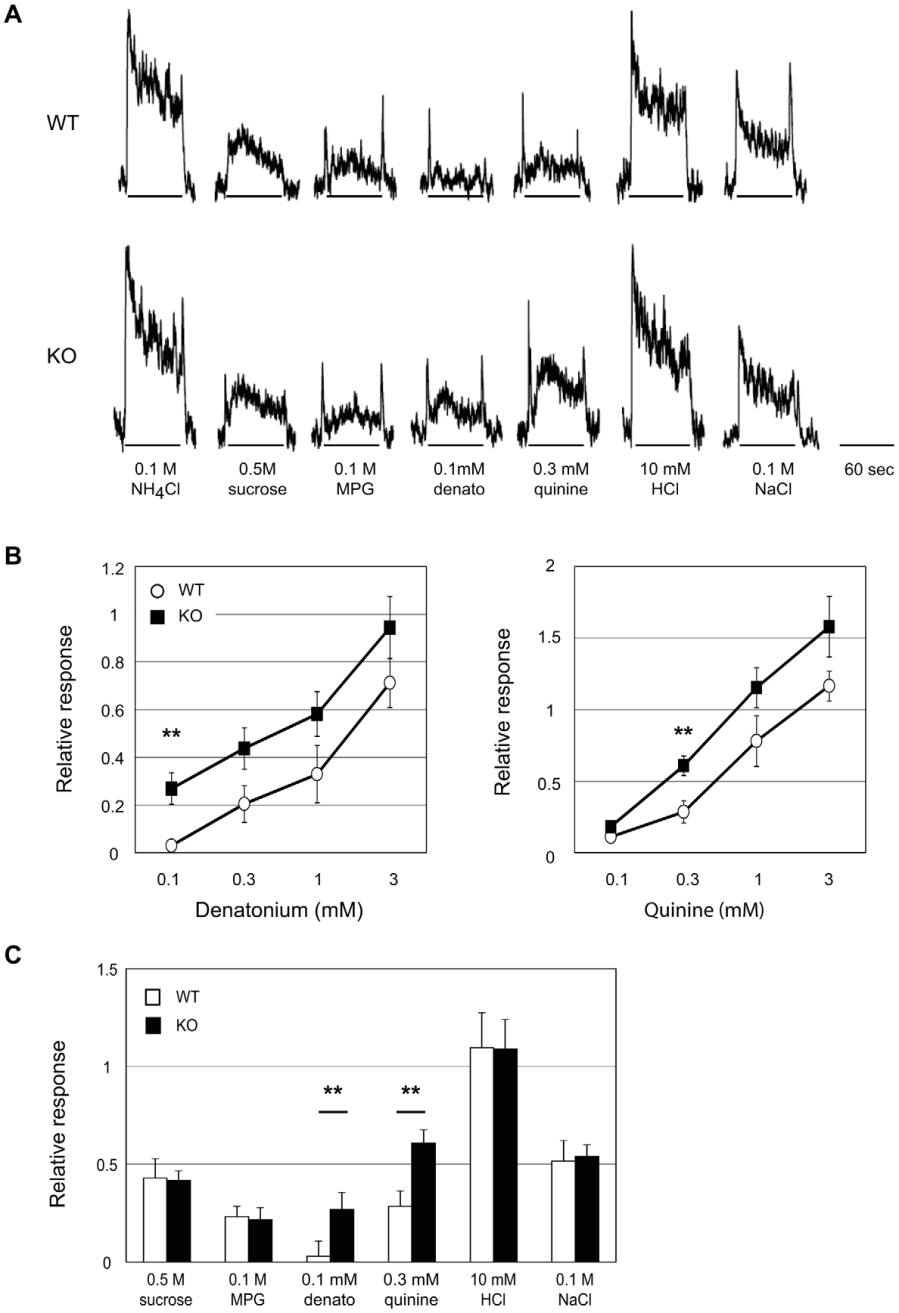
Whole-nerve recordings from the glossopharyngeal nerves of WT and Serca3 KO mice upon taste stimuli. (A) Representative responses of the glossopharyngeal nerves to the taste substances: sweet (0.5 M sucrose), umami (0.1 M MPG), bitter (0.1 mM denatonium (denato) and 0.3 mM quinine), sour (10 mM HCl), and salty (0.1 M NaCl). The responses to 0.1 M NH_4_Cl were used as reference for data analysis. The robust responses to bitter-tasting substances were recorded from the Serca3 KO mice versus WT mice. (B) Concentration–response curves to the bitter taste compounds denatonium (left) and quinine (right) indicated the significantly increased nerve responses to 0.1 mM denatonium and 0.3 mM quinine obtained from Serca3 KO mice (filled squares) compared with those of WT mice (open circles). (C) Integrated neural responses, such as those shown in (A), were normalized to the response elicited by 0.1 M NH_4_Cl. No significant difference in the nerve responses to sweet, umami, sour, and salty tastes is apparent between WT mice (open bars) and KO mice (filled bars). Data are means ± SEMs (n = 6–8). Mixed-model ANOVA and *post hoc t*-tests were performed (**p<0.01).

To determine whether the augmented behavioral responses of Serca3 KO mice to bitter compounds were attributable to the deficiency in the peripheral gustatory system, we performed electrophysiological recordings of the gustatory nerve with taste stimuli. We recorded from the glossopharyngeal nerve since it innervates taste buds in the posterior part of the tongue, in which the bitter taste receptors are more abundantly expressed [13]. Consistent with the behavioral data described above, the nerve responses in Serca3 KO mice to bitter taste stimuli (denatonium and quinine) were significantly increased compared with those of the WT mice (Fig. 7, Table S4). In contrast, we observed no significant differences between KO mice and WT mice in the nerve responses to sweet, umami, sour, and salty tastes (Fig. 7A and C, Table S4).

### Serca2 is co-expressed with T1R3

**Figure 8:**
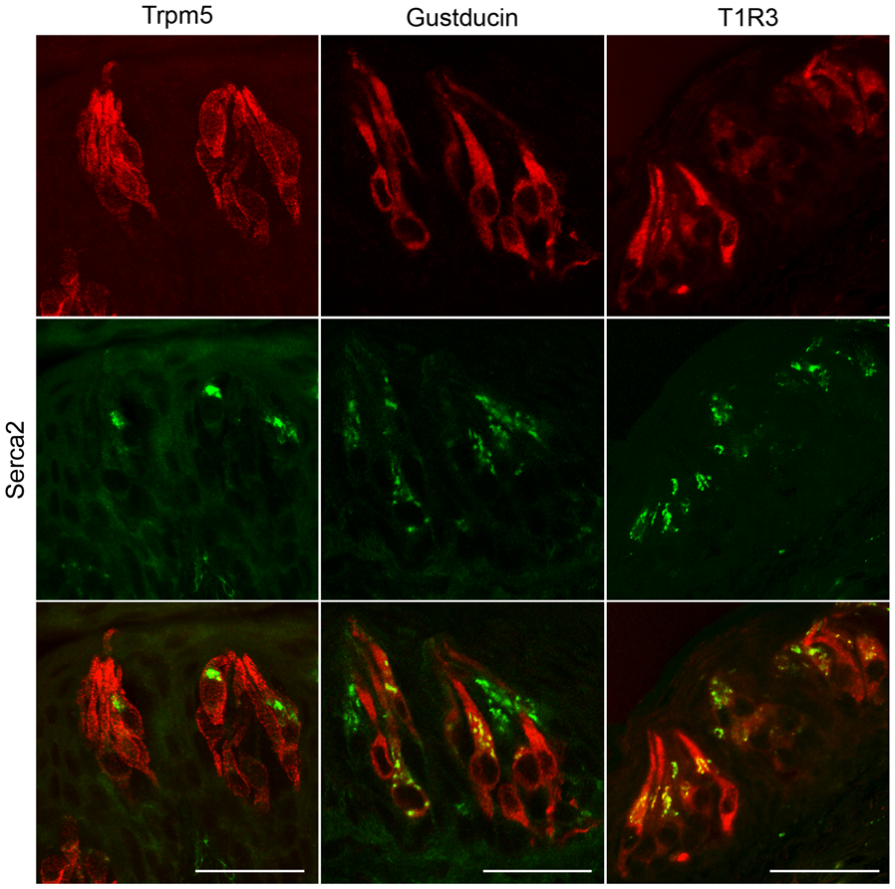
Serca2 is expressed in sweet and umami taste receptor cells. Rat circumvallate papilla sections were double immunostained with anti-Serca2 antibody (green) and with antibodies against Trpm5 (left panels), α-gustducin (middle panels), or T1R3 (right panels) (red). Overlay of the immunofluorescence images (bottom row) showed that Serca2 was expressed mainly in a subpopulation of Trpm5/T1R3-expressing cells, and, to a lesser extent, in α-gustducin-expressing cells. Scale bars, 25 µm.

To investigate the differential effect of *Serca3* gene nullification on the taste responses to bitter substances versus sweet and umami compounds, we reasoned that other functionally related proteins such as another member of the SERCA family might compensate in part for Serca3's absence in the KO mice. RT-PCR showed that *Serca2* is expressed in mouse lingual epithelia bearing CV and foliate taste papillae, but *Serca1* is not (data not shown). Immunocytochemical results indicated that, unlike immunostaining with Serca3 antibody, which was distributed throughout the taste cell body, Serca2 staining was punctuate and localized to limited cytoplasmic regions, in many cases the area apical to the nucleus (Fig. 8). To determine which types of taste cells express Serca2, we performed double-label immunohistochemistry on rat CV taste epithelia with antibody to Serca2 along with the antibodies recognizing Trpm5, α-gustducin, or T1R3 protein. Since the anti-Serca2 antibody was generated in mice, we used taste tissue sections from rat instead of mouse to eliminate possible intrinsic background staining in the mouse tissue. We found that (1) Trpm5 and Serca2 signals were largely overlapping, but a few cells expressed Trpm5 only; (2) many cells expressed both Serca2 and α-gustducin, but some cells expressed only one or the other; and (3) nearly all Serca2-expressing cells expressed T1R3 (Fig. 8). Together, these data indicate that Serca2 is expressed mainly in a subpopulation of Trpm5/T1R3-expressing cells, suggesting that it is likely to contribute to sweet and umami taste sensation.

## Discussion

In this study, we performed transcriptomic screen of single taste bud cells by subtracting cDNA of type I cells from that of TRCs to facilitate the isolation of genes specifically or more abundantly expressed in TRCs than in type I taste bud cells. Among these differentially expressed genes, we identified *Serca3*, a member of SERCA family that encodes a molecular pump known to actively transport Ca^2+^ from the cytosol into the ER. Three genes, *ATP2A1*, *2*, and *3*, encode SERCA 1, 2, and 3 transcripts, which produce various isoforms in a species-, tissue-, and developmental stage-specific manner [43]. In taste tissue of adult rodents, we used splice variant-specific PCR primers and detected transcripts for all known Serca3 variants: two rat variants (*Serca3a* and *3b/c*) and three mouse variants (*Serca3a*, *3b*, and *3c*). With the primers covering the entire coding sequences, we also isolated the transcripts for all variants except mouse *Serca3c*, and as the variant-specific PCR data (Fig. 1) indicated, the failure to amplify *Serca3c* full-length coding sequence was likely due to its low expression level in taste buds. The presence of multiple Serca3 isoforms may allow taste bud cells to precisely regulate their focal and/or global Ca^2+^ signals in a cell-specific manner. For example, a previous study showed that some human SERCA3 protein isoforms have distinct Ca^2+^ pumping properties but similar affinities toward Ca^2+^ [48]. Further investigation into the exact distribution of each Serca3 isoform among different types of taste bud cells can provide additional information on the fine control of Ca^2+^ signals in these cells.

Immunohistochemical studies with an antibody that recognizes all known Serca3 isoforms in mice showed that a subset of taste bud cells from all examined taste tissue—CV, foliate, and fungiform papillae and soft palate—express this Ca^2+^ pump, whereas extragemmal epithelial cells showed no detectable signals (Fig. 2). Further, Serca3-expressing taste bud cells are the same as those expressing Trpm5 but different from those expressing Gad1 (Fig. 3), indicating that Serca3 protein is expressed exclusively in sweet/umami/bitter TRCs but not in presynaptic cells.

Functionally, the activation of sweet, umami, and bitter taste receptors eventually leads to the Ca^2+^ release from the ER into the cytosol. The increased cytosolic Ca^2+^ opens the Trpm5 channel, resulting in the depolarization of the receptor membrane potential and release of the transmitters onto the afferent nerve fibers. The Serca3 pump may not be active in the resting TRCs because the apparent Ca^2+^ affinity of Serca3 is low, with a K_0.5_ of ∼1.1 µM, and the normal basal [Ca^2+^]_c_ is about 0.1 µM, well below its K_0.5_ [37], [48], [49], [50]. Once activated by the rising [Ca^2+^]_c_, however, Serca3 has a high uptake rate [49], and is able to sequester Ca^2+^ back into the ER, which modulates the net amount of [Ca^2+^]_c_ and affects downstream signal transduction. The events also could lead to the termination of taste signal transduction. The complete co-expression of Serca3 and Trpm5 in TRCs suggests that Serca3 functions to reverse increased [Ca^2+^]_c_ induced by sweet, umami, and bitter taste-receptor-mediated PLCβ2-IP_3_R3 signaling. The sequestration of Ca^2+^ also enables TRCs to maintain a constant net total amount of Ca^2+^ within a cell (cytoplasm+cellular organelle) over a long period. This mechanism seems to differ from that used by presynaptic cells, which depend on Ca^2+^ influx to initiate synaptic transmission and utilize Na^+^/Ca^2+^ exchangers to transport Ca^2+^ across the plasma membrane back into the extracellular space [38], [39].

To demonstrate for the first time how malfunction of the Ca^2+^ clearance mechanism affects taste perception, we analyzed the behavioral responses of mice lacking Serca3 to tastants representative of five primary taste qualities (Figs. 5 and 6). The deficiency of Serca3 did not affect the animals' preferences for various concentrations of sour and salty stimuli, which agrees with our immunostaining data showing no expression of Serca3 in presynaptic cells or in cells other than TRCs. In tests with sweet, umami, and bitter taste stimuli, we observed significant differences between the KO and WT mice in aversive responses to the bitter taste substances denatonium (Figs. 5D and 6C) in both the 48-h two-bottle preference assays and brief-access tests and to quinine in the brief-access tests (Fig. 5E). Although statistically insignificant, the KO mice appeared to display an increased aversion to quinine as well, compared with WT mice (Fig. 6D) in brief-access tests.

Electrophysiological data from the gustatory nerve responses indicate that the altered behavioral responses to bitter taste in Serca3 KO mice are contributable to the deficiency in the peripheral taste system (Fig. 7). Electrical recordings from the glossopharyngeal nerve that innervates the taste buds with relatively abundant expression of bitter taste receptors showed significantly stronger responses for bitter taste compounds in Serca3 KO mice than in WT control mice, whereas the responses to sweet, umami, salty, and sour substances were indistinguishable between these two groups of mice.

Intriguingly, the *Serca3* gene deletion seemed to have no significant effect on preference or gustatory nerve responses for sweet and umami tastes (Figs. 5–7). We observed a trend toward stronger preferences in the KO mice for sweet and umami taste substances in behavioral tests, suggesting a perceptible but minor contribution of Serca3 in these tastes. To address the disparity in the behavioral responses of the Serca3 KO mice to bitter versus sweet and umami taste substances, we examined the expression of Serca2 in taste tissue (Fig. 8). Double immunolabeling of rat CV taste buds showed that Serca2 is expressed in a subset of Trpm5-expressing cells and is almost completely co-expressed with T1R3 in the same population of cells. Previous studies have shown that Trpm5 is present in all sweet, umami, and bitter TRCs, while T1R3 occurs only in sweet and umami TRCs. Thus, our results suggest that Serca2 is present in sweet and umami TRCs and is likely absent in the bitter receptor cells. Double-immunostaining data with antibodies against Serca2 and α-gustducin also support this conclusion: the cells expressing these two proteins only partially overlap (Fig. 8), since α-gustducin is present in most bitter taste receptor cells but in only a small fraction of T1R3-expressing cells in rodent CV papillae [13], [51], [52]. Therefore, we speculate that the absence of significant alterations in behavioral and nerve responses to sweet and umami taste stimuli in Serca3 KO mice can be attributed to Serca2, which compensates for the lack of Serca3 function in mutant sweet and umami TRCs.

From our data, we propose the following model. First, bitter taste stimuli activate the taste receptor-G protein-PLCβ2-IP_3_R3 signaling cascade, as well described previously [2], [17], [20]. Upon activation, IP_3_R3 releases Ca^2+^ into the cytoplasm from the ER, where Serca3 is localized, which leads to an increase of [Ca^2+^]_c_, especially in the immediate vicinity of the ER in a short time. This increased [Ca^2+^]_c_ activates Serca3, which begins Ca^2+^ uptake into the ER, causing a net decrease in the amount of cytoplasmic Ca^2+^ that can be used to activate the downstream signaling molecules, such as Trpm5, thus modulating the activity of the downstream taste signaling cascade [12], [21]. In Serca3 deficient bitter TRCs, without such a decrease in [Ca^2+^]_c_, higher levels of Ca^2+^ may be available for the downstream signaling cascade. The process can increase transmitter release onto the nerve fibers and enhance bitter taste sensation, which in turn engenders a stronger aversive response to the tastants. The stronger aversion to these bitter compounds by the KO mice clearly demonstrates the contribution of a Ca^2+^ clearance mechanism in animal taste perception. In sweet and umami TRCs, both Serca2 and Serca3 can be involved in cytoplasmic Ca^2+^ uptake. And Serca2 may partially compensate the role of Serca3 in these cells, leading to statistically insignificant but perceptible increases in the responses of the KO mice to these palatable taste stimuli.

Further studies on taste bud cell responses upon taste stimuli can quantitatively characterize the effect of Serca3 mutation on kinetics of calcium responses in taste bud cells and its correlation with the altered gustatory nerve responses and, eventually, with the perception and behavioral responses. Unlike other KO mouse models such as T1R3 or Trpm5 KO that abolish nearly all responses to specific taste qualities [12], [17], [53], [54], Serca3 KO mice provide a distinct model with a gradual change in taste reception. Additional data from such mouse models may further our understanding of how the central nervous system translates qualitative and quantitative sensory information into perception.

Our results illustrate that despite many commonalities in the sweet, umami, and bitter taste signaling pathways, different Ca^2+^ clearance/taste signal regulation mechanisms may be used by the TRCs for these three tastes. This notion is supported by a recent report describing the possible role of another calcium-signaling-related protein, calcium- and integrin-binding protein 1 (CIB1), in sweet TRCs [55].

It is also noteworthy that most of Serca2 proteins appeared to be located in the ER regions closer to the taste pore relative to the nucleus, which may have functional implications. It is possible that the proximately situated pumps can more rapidly respond to the signaling cascades initiated by the activated taste receptors at the taste pore. The exact contribution of Serca2 to taste signal processing in taste bud cells can be further revealed by studies with Serca2 KO mice. However, the systemic knockout of this gene turned out to be embryonic lethal [56]. Generation and characterization of a taste-cell-specific Serca2 KO mouse can further define Serca2's function in taste perception and may provide novel insights into taste signal regulation mechanisms.

In summary, our findings show that sweet, umami, and bitter TRCs possess different Ca^2+^ clearance machineries and that mutations in these proteins can alter taste perception.

## Materials and Methods

### Animals

FVB/NJ (WT) mice and CB6-Tg(Gad1-EGFP)G42Zjh/J transgenic mice (referred to as Gad1-GFP mice throughout) were purchased from the Jackson Laboratory (Bar Harbor, ME). Serca3 knockout (KO) mice [47] and Trpm5-GFP transgenic mice [40] were bred and maintained at the Monell Chemical Senses Center animal facility. Trpm5-GFP transgenic mice carry a green fluorescent protein (GFP) transgene driven by Trpm5 (transient receptor potential cation channel, subfamily M, member 5) promoter. All procedures involving animals were approved by the Monell Chemical Senses Center Institutional Animal Care and Use Committee.

### Construction of the subtracted single-taste-cell library

To isolate the genes that are selectively or preferentially expressed in sweet, umami, or bitter taste receptor cells (TRCs), we generated rat subtracted single-TRC cDNA libraries. First, we performed single-taste-cell transcriptome amplification as described previously [18]. Briefly, a rat tongue was removed, and Tyrode's solution containing 2 mg/ml dispase II and 1 mg/ml collagenase was subepithelially injected into the tongue, which was then incubated at 25°C for 30 min. The lingual epithelium was peeled off from the rest of the tongue, and circumvallate (CV) and foliate taste papillae were excised and dissociated into individual cells. Taste bud cells were identified based on their bipolar shape and individually transferred into 0.5-ml tubes. First-strand cDNA was synthesized with reverse transcriptase (Invitrogen, Carlsbad, CA) and oligo(dT) primers, tailed with dATP and terminal transferase (Roche Applied Science, Indianapolis, IN), and then amplified with oligo(dT) primers. Aliquots of the amplified products were used for *post hoc* cell typing by quantitative real-time PCR (qPCR) with Power SYBR Green Master Mix (Applied Biosystems, Foster City, CA) and specific PCR primers for known cell-type marker genes (see Table S1): ectonucleoside triphosphate diphosphohydrolase 2 (*Entpd2*) for type I cells [4]; *Trpm5* and *Tas1r1* and *Tas1r2* (taste receptor, type 1, member 1 and member 2) for all sweet/umami/bitter-, umami-, and sweet-sensing TRCs (or type II cells), respectively [9], [10], [12]; and *Snap25* (synaptosomal-associated protein 25) for presynaptic (or type III) cells [57].

We then subtracted the amplified products of *Entpd2*-expressing type I taste bud cells from those of a single cell expressing *Trpm5* using a previously published method, with some modification [58]. Briefly, the driver cDNA pooled from rat type I cells was labeled with an equal amount of photobiotin (Sigma, St. Louis, MO) by ultraviolet irradiation in a Stratalinker 2400 crosslinker (Stratagene, Cedar Creek, TX) and then hybridized with tracer cDNA from a single TRC at a ratio of 24∶1 (cDNA amount of driver vs. tracer). The mixture was incubated in the hybridization buffer (12.5% PEG8000, 1.5 M NaCl, 10 mM EPPS, pH 8.25, 1 mM EDTA, pH 8.0, and 0.1% SDS) according to the following parameters: 5 min at 80°C, ramp 80°C to 68°C for 16 min, 90 min at 68°C, 10 min at 80°C. The hybrids were then removed with streptavidin and purified with phenol/chloroform extraction. After two rounds of subtraction, the resultant cDNA was further amplified using restriction-enzyme site-tagged oligo(dT) primers and then subcloned into the pBluescript II vector. The sequences of the insert DNA were determined by DNA sequencing analysis and BLAST-searched against the GenBank databases.

### Isolation and expression analysis of Serca3 cDNA

One clone, A14, matched part of the Serca3 cDNA sequence (GenBank accession number NM_012914.1) [42]. To determine whether Serca3 is selectively expressed in taste tissue, rat and mouse lingual epithelia containing CV and foliate papillae or devoid of any taste buds as negative control were excised, and total RNA was prepared using Absolutely RNA® Miniprep Kit (Stratagene), which was reverse transcribed into first-strand cDNA using random primers and the Superscript III kit (Invitrogen). PCR amplification was performed with primers that covered the full coding regions of mouse and rat Serca3 transcripts (see Table S2A). To ensure detection of all expressed splice variants, additional PCR reactions were performed using primers specific for all known variants (see Table S2B). The PCR products were subcloned into pGEM-T Easy (Promega, Madison, WI) or pCR4-TOPO vector (Invitrogen), and the insert DNA was sequenced with the vector primers.

### Immunohistochemistry

Mouse and rat tongue and soft palate were removed and fixed with 4% paraformaldehyde in phosphate-buffered saline (PBS) for 1 h on ice and cryoprotected in 20% sucrose in PBS at 4°C for 2 h and then in 30% sucrose in PBS at 4°C overnight. The regions containing taste papillae (fungiform on the dorsal surface of anterior one-third of the tongue, CV on the dorsal surface of posterior tongue, foliate at the lateral edge of posterior tongue, and soft palate) were excised and embedded in Tissue-Tek® OCT compound (Sakura Finetek USA, Inc., Torrance, CA). Frozen sections (10 µm thickness) were obtained using a cryostat and were then permeabilized with PBS-T (0.3% Triton X-100 in PBS) and blocked with the blocking buffer (2% goat serum, 3% BSA, 0.3% Triton X-100, and 0.1% sodium azide in PBS) overnight at 4°C. The sections were incubated with properly diluted primary antibodies at 25°C for 4 h or at 4°C overnight. After six washes with PBS-T for 10 min each, the sections were incubated with secondary antibodies at 25°C for 1 h. The working concentrations and other information about all antibodies used in this study are summarized in Table S3. The sections were thoroughly washed with PBS-T again before being covered with Vectashield H-1000 (Vector Laboratories, Burlingame, CA). Fluorescent images were taken using a confocal microscope (Leica Microsystems Inc., Bannockburn, IL). Control experiments performed without primary antibodies, or with taste tissue sections from Serca3 KO mice, showed neither nonspecific staining of primary antibodies nor cross-reactivity between secondary antibodies, validating antibody specificity. We verified the reproducibility of the results by performing immunostaining with at least 40 taste tissue sections from five each of WT and Serca3 KO mice, with total 20 sections from at least 2 mice from each Trpm5-GFP and Gad1-GFP transgenic line, and with 30 taste tissue sections from three rats for Serca2 immunostaining experiments.

### Taste solutions

Taste solutions were prepared in deionized water using reagent-grade chemicals purchased from Sigma-Aldrich. All taste stimuli were presented at room temperature.

### Two-bottle taste preference tests

Sex- and age-matched Serca3 KO and WT control (FVB/NJ) mice (9–10 for each group, except 5 KO mice for NaCl tests) were used for two-bottle taste preference tests as previously described [19], [59]. The animals were 7–15 weeks old at the start of each experiment. The mice were given access for 48 h to two bottles, one containing deionized water and the other a tastant solution, and to food ad libitum. After 24 h, intake of each fluid was recorded and the bottle positions were switched to minimize any positional effect. After another 24 h, fluid intake was again recorded. Mice were then given two bottles of deionized water for at least 4 days between different tastant trials.

Each tastant was presented in an ascending concentration series: sweet, 5, 10, 20, 40, and 80 mM sucrose (caloric) and 0.1, 0.3, 1, 3, and 10 mM sodium saccharin (noncaloric artificial sweetener); umami, 0.1, 0.3, 1, 3, and 10 mM monosodium L-glutamate (MSG); bitter, 0.01, 0.03, 0.1, 0.3, 1, and 3 mM denatonium benzoate and 0.01, 0.03, 0.1, 0.3, and 1 mM quinine hydrochloride; sour: 0.1, 0.5, 1, and 2.5 mM citric acid; salty: 37.5, 75, 150, and 300 mM NaCl.

### Brief-access tests

Sex- and age-matched Serca3 KO and WT control (FVB/NJ) mice (7 for each group) were used for these tests. The animals were 5–7 weeks old at the onset of each experiment. The licking rate of individual mouse presented with a series of different tastants was measured using a Davis MS-160 gustometer (DiLog Instruments, Tallahassee, FL) as previously described [60]. Test stimuli consisted of serial concentrations of: sodium saccharin (0.1, 1, and 10 mM); MSG (1, 3, 10 mM) in the presence of 100 µM amiloride and 1 mM inosine monophosphate (IMP); denatonium benzoate (0.1, 0.3, and 1 mM); quinine hydrochloride (0.05, 0.25, and 1 mM); citric acid (1, 3, and 10 mM); and NaCl (0.1, 0.3, and 0.5 M). In order to measure responses to attractive stimuli (saccharin and MSG), mice were fed 1 g food and 1.5 ml water over the 24 h before testing and were tested every second day. For aversive stimuli (denatonium, quinine, citric acid, and NaCl), mice were placed on a 22-h restricted water-access schedule and were tested daily. To ensure adequate sampling, a maximum of five different solutions (including a water control) were presented during a test. Stimulus presentation order was randomized within blocks. The duration of each trial (5 s) was regulated by a computer-controlled shutter that allowed access to the sipper tube with a 10 s inter-presentation interval. A water rinse presentation (1 s) was interspersed between the test trials for the normally avoided stimuli to help control for potential carry-over effects as previously described [61].

### Data analysis

Data from the two-bottle preference tests are presented as percent preference scores, which were calculated using average daily fluid intakes of individual mice: [solution intake/total fluid (solution+water) intake]×100. A preference score of 50% indicates neither preference nor avoidance of the solution compared with water; a score >50% suggests preference for the solution, and <50% avoidance of the solution, compared with water. For brief-access tests, the average number of licks per trial for each stimulus concentration was divided by the average number of water licks per trial, yielding a tastant/water lick ratio.

Preference scores (two-bottle tests) and tastant/water lick ratio (brief-access tests) were analyzed using a mixed-model analysis of variance (ANOVA), and genotype (between-subjects) and tastant concentration (within-subjects) effects were evaluated. If there was a significant effect, then *post hoc* pairwise comparisons of means at each test solution concentration were conducted with Student *t*-tests between genotype groups (two-tailed unpaired distribution, except for NaCl tests in two-bottle tests, for which a two-sample equal variance was used). All statistical analyses were performed using Microsoft Excel software and the software package Statcel (OMS, Tokyo, Japan). Statistical rejection was set at the 0.05 level for all data analysis.

### Gustatory nerve recordings

The procedures for the mouse glossopharyngeal (GL) nerve recordings were described previously [62]. Briefly, Serca3 KO and WT control (FVB/NJ) mice (6–8 for each group) were anesthetized with an intraperitoneal injection of sodium pentobarbital (40–50 mg/kg of mouse body weight) and maintained at a surgical level of anesthesia with additional injection of sodium pentobarbital. The trachea of each animal was cannulated, and the mouse was then fixed in the supine position with a head holder. The right GL nerve was exposed by removal of the digastric muscle and posterior horn of the hyoid bone and then dissected free from underlying tissues and cut near its entrance to the posterior lacerated foramen. For whole-nerve recording, the entire nerve was placed on a platinum wire recording electrode. An indifferent electrode was positioned nearby in the wound. Neural responses induced by chemical stimulations of the tongue were fed into an amplifier (Grass Instruments, West Warwick, RI), monitored on an oscilloscope and audiomonitor. Whole nerve responses were integrated with a time constant of 1.0 sec and recorded on a computer for later analysis using PowerLab system (AD Instruments, Colorado Springs, CO).

The solutions used to stimulate the posterior part of the tongue were as follows: sucrose, 0.5 M; mono potassium glutamate (MPG), 0.1 M; denatonium, 0.1, 0.3, 1, and 3 mM; quinine, 0.1, 0.3, 1, and 3 mM; HCl, 10 mM; and NaCl, 0.1 M. The compounds were applied to the tongue for 1 min, followed by rinse with deionized water.

In the data analysis for whole-nerve response to each stimulus, the magnitude of the integrated response at 10, 15, 20, 25, 30, 35, 40, 45, and 50 s after stimulus onset was measured and averaged. Relative response magnitudes (averaged) for each test stimulus were normalized to the response magnitude to 0.1 M NH_4_Cl. Genotype differences in responses to compounds tested at single concentrations were assessed using *t*-tests. Responses to compounds tested at multiple concentrations were analyzed using a mixed-model ANOVA assessing effects of genotype (between-subjects factor) and concentration (within-subjects factor). If significant effects were detected using ANOVA, then planned comparison or *post hoc t*-tests were used to compare strain means for specific concentrations. For all tests, *P*<0.05 was considered significant.

## Supporting Information

Table S1 **Primers for cell type-specific marker genes used in cell typing qPCR.**
(DOC)

Table S2 **Primers for Serca3 used in RT-PCR.**
(DOC)

Table S3 **Antibodies.**
(DOC)

Table S4 **ANOVA results for the behavioral tests and the gustatory nerve recordings to taste compounds (WT vs. Serca3 KO mice).**
(DOC)
