# Supplementary material for: Sarco/Endoplasmic Reticulum Ca2+-ATPases (SERCA) Contribute to GPCR-Mediated Taste Perception
Source: PLoS One. 2011 Aug 2;6(8):e23165. doi: 10.1371/journal.pone.0023165 (PMC3149081; doi:10.1371/journal.pone.0023165)
Supplement: Table S1 — Primers for cell type-specific marker genes used in cell typing qPCR. (DOC) [file pone.0023165.s001.doc]

**Table S1. Primers for cell type-specific marker genes used in cell typing qPCR**

| Gene ID  (Accession number) | Orientation | Sequence | Product size (bp) | Cell type |
| --- | --- | --- | --- | --- |
| *Entpd2/NTPDase2*  (NM_172030) | Forward | gtgaactgggagcctctcccaactc | 280 | I |
|  | Reverse | gcctgtaggctctggtgcctgcctt |  |  |
| *Trpm5*  (XM_344979) | Forward | agggacagtgaggaggaggt | 220 | II  TRCs |
|  | Reverse | ctagcagaggctggctgact |  |  |
| *Tas1r1*  (XM_342986) | Forward | ttcagcctgctcctcaactt | 170 | II  umami TRCs |
|  | Reverse | cggcagagaatcacatagca |  |  |
| *Tas1r2*  (AF127390) | Forward | caacgaagccaagttcatca | 255 | II  sweet TRCs |
|  | Reverse | ctcatggtgtagccctggat |  |  |
| Snap25  (NM_030991) | Forward | actacgcatgctcagtattgggaca | 330 | III  presynaptic |
|  | Reverse | accacagggagatggtgatcaaca |  |  |
