# Supplementary material for: Sarco/Endoplasmic Reticulum Ca2+-ATPases (SERCA) Contribute to GPCR-Mediated Taste Perception
Source: PLoS One. 2011 Aug 2;6(8):e23165. doi: 10.1371/journal.pone.0023165 (PMC3149081; doi:10.1371/journal.pone.0023165)
Supplement: Table S2 — Primers for Serca3 used in RT-PCR. (DOC) [file pone.0023165.s002.doc]

**Table S2. Primers for Serca3 used in RT-PCR**

A. Primers used to clone the full length of the coding sequence

| Species | Amplifying region | Orientation | Sequence | Product size (bp) |
| --- | --- | --- | --- | --- |
| Rat | 5’ part | Forward | cctcaagttcgcagcattctgcac | 1546 |
|  |  | Reverse | gtcccgggagaattcgaggg |  |
|  | Center part  (5’ side) | Forward | gcagtgggagacaaggtacc | 1583 |
|  |  | Reverse | ggttccacacgggcaaagcatcg |  |
|  | Center part  (3’ side) | Forward | gtggctgcctgtatcacacgctg | 961 |
|  |  | Reverse | gtggcatacgcagcagtgac |  |
|  | 3’ part  (*Serca3a*-specific) | Forward | gtaggcctggctacagtggc | 1623 |
|  |  | Reverse | gggacttttgctaccaggtg |  |
| Mouse | *Serca3a-c* | Forward | ccggaggagaagcgacctgga | a: 3138  b: 3211  c: 3224 |
|  |  | Reverse | gtggctgagtgtggaggcag |  |

B. Alternative spliced variant-specific primers

| Species | Variant name  (Accession number) | Orientation | Sequence | Product size (bp) |
| --- | --- | --- | --- | --- |
| Rat | *Serca3a*  (NM_012914.1) | Forward | gcctcttaaccctgctgttg | 171 |
|  |  | Reverse | gggacttttgctaccaggtg |  |
|  | *Serca3b/c*  (AF458230.1) | Forward | tatgcaggcttggtgtaagc | 202 |
|  |  | Reverse | ccaaagcaggtcttagctagc |  |
| Mouse | *Serca3a*  (NM_001163336.1) | Forward | cacatggatgaaaaaaagg | 232 |
|  | *Serca3b*  (NM_016745.3) | Forward | cccataccacaccggaaaaaaagg | 237 |
|  | *Serca3c*  (NM_001163337.1) | Forward | cacaccgggttggcttcttgga | 242 |
|  | *Serca3a-c* | Reverse | cagagcagtagggagggaca | - |
