# Supplementary material for: Sarco/Endoplasmic Reticulum Ca2+-ATPases (SERCA) Contribute to GPCR-Mediated Taste Perception
Source: PLoS One. 2011 Aug 2;6(8):e23165. doi: 10.1371/journal.pone.0023165 (PMC3149081; doi:10.1371/journal.pone.0023165)
Supplement: Table S3 — Antibodies. (DOC) [file pone.0023165.s003.doc]

**Table S3. Antibodies**

A. Primary antibodies (commercial)

| Antigen Name | Host | Class | Working dilution | Vendor | Catalog number |
| --- | --- | --- | --- | --- | --- |
| SERCA3 | Rabbit | Polyclonal | 1:1500 | Pierce Biotechnology  (Rockford, IL) | PA1-910A |
| α-gustducin | Rabbit | Polyclonal | 1:2000 | Santa Cruz  (Santa Cruz, CA) | SC-395 |
| PLCβ2 | Rabbit | Polyclonal | 1:2000 | Santa Cruz | SC-206 |
| NCAM | Rabbit | Polyclonal | 1:1000 | Millipore  (Billerica, MA) | AB5032 |
| DDC | Rabbit | Polyclonal | 1:500 | GeneTex  (Irvine, CA) | GTX30448 |
| SERCA2 | Mouse | Monoclonal | 1:2500 | EMD Chemicals Inc. (Princeton, NJ) | 564702 |

B. Primary antibodies (non-commercial)

| Antigen Name | Host | Class | Working dilution | Immunogen | Reference |
| --- | --- | --- | --- | --- | --- |
| TRPM5 | Rabbit | Polyclonal | 1:2500 | A peptide of mouse Trpm5  (residues 1028-1049) | [46] |
| T1R3 | Rabbit | Polyclonal | 1:500 | A peptide of mouse T1R3 (residues 239-255) | [52] |

C. Secondary antibodies

| Antigen Name | Label | Host | Class | Working dilution | Vendor | Catalog number |
| --- | --- | --- | --- | --- | --- | --- |
| Rabbit IgG (H+L) | DyLight549 | Goat | Polyclonal | 1:5000 | Rockland Immunochemicals  (Gilbertsville, PA) | 611-142-122 |
| Mouse IgG | Alexa FluorR 488 | Goat | Polyclonal | 1:1000 | Invitrogen | A-11001 |
