# Supplementary material for: Sarco/Endoplasmic Reticulum Ca2+-ATPases (SERCA) Contribute to GPCR-Mediated Taste Perception
Source: PLoS One. 2011 Aug 2;6(8):e23165. doi: 10.1371/journal.pone.0023165 (PMC3149081; doi:10.1371/journal.pone.0023165)
Supplement: Table S4 — ANOVA results for the behavioral tests and the gustatory nerve recordings to taste compounds (WT vs. Serca3 KO mice). (DOC) [file pone.0023165.s004.doc]

**Table S4. ANOVA results for the behavioral tests and the gustatory nerve recordings to taste compounds (WT vs. Serca3 KO mice)**

|  | **Two-bottle preference test** | | **Brief-access test** | | **Gustatory nerve recording** | |
| --- | --- | --- | --- | --- | --- | --- |
| **Tastant** | ***df*** | ***F* value** | ***df*** | ***F* value** | ***df*** | ***F* value** |
| Sucrose | 1,16 | 2.1 | N.D. | N.D. | N.D. | N.D. |
| Saccharin | 1,18 | 0.7 | 1,14 | 0.8 | N.D. | N.D. |
| MSG | 1,16 | 1.5 | N.D. | N.D. | N.D. | N.D. |
| MSG+IMP | N.D. | N.D. | 1,14 | 0.7 | N.D. | N.D. |
| Denatonium | 1,18 | 8.7** | 1,12 | 10.2** | 1,14 | 5.2* |
| Quinine | 1,16 | 7.2* | 1,12 | 0.7 | 1,14 | 6.0* |
| Citric acid | 1,18 | 0.3 | 1,12 | 0.4 | N.D. | N.D. |
| NaCl | 1,13 | 0.1 | 1,12 | 0.6 | N.D. | N.D. |

Preference scores from the two-bottle preference tests (Fig. 5), tastant/water lick ratios from the brief-access tests (Fig. 6), and response magnitudes from the gustatory nerve recordings (Fig.7B) were analyzed by a mixed-model ANOVA as described in the Methods. *df*: degrees of freedom. N.D. not determined. **P* < 0.05, ***P* < 0.01.
